# The role of cyclooxygenase-2 (COX-2) and inflammatory markers in the progress of Alport syndrome in Egyptian children

**DOI:** 10.1186/s12887-025-05412-2

**Published:** 2025-01-24

**Authors:** Moushira Zaki, Hisham A. Orban, Mohamed A. Shahba, Rehab S. I. Moustafa, Ahmed Adel, Fatina I. Fadel, Abeer Selim, Hala T. El-Bassyouni, Eman R. Youness

**Affiliations:** 1 Biological Anthropology Department, Medical Research and Clinical Studies Institute, National Research Centre, Cairo, Egypt; 2 Medical Biochemistry Department, Medical Research and Clinical Studies Institute, National Research Centre, Cairo, Egypt; 3 Child Health Department, Medical Research and Clinical Studies Institute, National Research Centre, Cairo, Egypt; 4 Department of Pediatrics, Pediatric Nephrology Unit. Kasr Al-Ainy Faculty of Medicine, Cairo University, Cairo, Egypt; 5 Department of Pediatrics, Medical Research and Clinical Studies Institute, National Research Centre, Cairo, Egypt; 6 Clinical Genetics Department, Human Genetics and Genome Research Institute, National Research Centre, Cairo, Egypt

**Keywords:** Alport syndrome, Cyclooxygenase-2, Interleukin 4, Plasminogen activating inhibitor 1, Prostaglandin E2

## Abstract

**Background:**

Chronic inflammation and its control are crucial to the responses of glomerular and renal tubular cells. This contributes to the pathogenic mechanisms and advancement of the disease in Alport syndrome. The study aimed to elucidate the role of cyclooxygenase-2, Interleukin 4, Plasminogen activating inhibitor 1, and Prostaglandin E2 in the development and course of Alport syndrome.

**Methods:**

In our study inflammatory markers were evaluated in 26 Alport syndrome patients, 15 males and 11 females and 24 controls.

**Results:**

Their age ranged from 4 to 16 years (mean ± SD was 8.50 ± 2.877) and 24 were normal controls matching age and sex. The serum levels of cyclooxygenase-2, Prostaglandin E2, Interleukin 4, and Plasminogen activating inhibitor 1 were evaluated in all patients. The serum level of cyclooxygenase-2, Prostaglandin E2, Interleukin 4, and Plasminogen activating inhibitor 1 were all increased significantly in the Alport syndrome patients compared to control (588.68 ± 73.08, 42.57 ± 4.18, 42.32 ± 3.49, and 846.47 ± 45.433, respectively versus controls (369.12 ± 50.28, 25.52 ± 4.98, 28.89 ± 3.19, and 312.79 ± 40.53 respectively).

**Conclusion:**

The role of inflammatory markers cyclooxygenase-2, Prostaglandin E2, Interleukin 4, and Plasminogen activating inhibitor 1 in Alport syndrome that are causally connected and have a role in the development and course of Alport disease was delineated. This may highlight and speculate an innovative strategy for targeting the creation of safe and efficient anti-inflammatory treatments to inhibit disease progression in Alport syndrome.

## Background

Alport syndrome is a type IV collagen hereditary disorder characterized by thin basement membrane nephropathy and focal segmental glomerulosclerosis. It is considered that one in every 50,000 live newborns has Alport syndrome [1]. The prevalence of Alport syndrome has been increasing as life expectancy has increased and awareness of the condition has improved. In Alport syndrome, the filters become inflamed which can lead to kidney failure [2].

The molecular features of Alport syndrome and thin basement membrane nephropathy emerge from mutations in the COL4A3, COL4A4, and COL4A5 genes, which lead to pathophysiologic consequences, such as chronic renal inflammation [3].

These named genes code the proteins that form collagen type IV of the glomerular basement membrane (GBM). The heterodimer COL4A3A4A5 forms the main part of the GBM, and it is crucial for the standard function of the glomerular filtration barrier (GFB). Changes in collagen type IV constituents cause disturbance of the GMB structure, causing leakage of red blood cells and albumin into the urine, and changing the architecture of the GFB, triggering and promoting inflammation and fibrosis, thus resulting in kidney damage and loss of renal function [4].

Recent research denotes that chronic inflammation and its regulation through proinflammatory molecules and anti-inflammatory factors is crucial for the response of renal tubular and glomerular cells to fibrosis [3].

Cyclooxygenase (COX-2) is necessary for prostaglandins, which are essential for normal kidney function. The regulation of renin release, inflammation, preservation of salt and water balance, renal vasodilation, attenuation of vasoconstriction, and prenatal renal development are all linked to these prostaglandins [5]. The role of COX-2 in kidney function is supported by the regulation of COX-2 expression by the renin-angiotensin system, glucocorticoids or mineralocorticoids, and aldosterone [6]. PGE2 and COX-2 mRNA, protein, and enzyme activity are elevated during renal failure. Furthermore, elevated blood pressure, decreased urine osmolarity, increased urine volume, salt, and protein are linked to variations in COX-2 expression [7]. Glomerular hypertension and renal tubular inflammation which exist in Alport syndrome are examples of intrarenal mechanisms that may be accountable for the upregulation of COX-2 expression, indicating intriguingly a potential involvement of COX-2 activation in the development and course of inflammation and nephropathies [3].

COX-2 inhibitors work by blocking the production of prostaglandins, which help regulate kidney function. In healthy kidneys, prostaglandins help to maintain blood flow to the glomeruli. However, in Alport syndrome, where the glomeruli are already inflamed, blocking the prostaglandin production with COX-2 inhibitors may further reduce blood flow and worsen kidney function [8].

IL-4 has a role in the modulation of the pro-inflammatory response and the development of renal tubule-interstitial injury in Alport syndrome [9]. In Alport syndrome, immunological modulation with interleukin-4, costimulatory blocking of T-cell activation, or suppression of macrophage migration may play a role in Alport syndrome treatment [10].

Plasminogen activating inhibitor 1 is a fibrogenic cytokine that accelerates the development of renal disorders. Plasminogen-activating inhibitor 1 plays a crucial part through different methods in the pathophysiology of renal interstitial fibrosis. It causes the tubular basement membrane to be destroyed by inducing the production of the matrix metalloproteinase-9 (MMP-9) gene in renal interstitial fibroblasts [11].

Prostaglandin E2 is the most abundant prostaglandin synthesized in the kidney. It is intricate in various pathophysiological and physiological processes in the kidney, including renal fibrosis a major pathological characteristic of progressive kidney diseases [12].

We have inclusion criteria for diagnosis of Alport syndrome and we aimed to use these markers to follow the inflammatory process and progress of the disease.

In this study, we aimed to elucidate the role of cyclooxygenase-2, Interleukin 4, Plasminogen activating inhibitor 1, and Prostaglandin E2 in the development and course of Alport syndrome. It was an important challenge as this is the first study on these Biomarkers effect on Alport syndrome and various renal diseases either Pediatric or adult. This addresses a scientific gap between research studies and a major physiological and pathological facts of the effect of Biomarkers on Alport syndrome and kidney diseases.

## Subjects and methods

The study comprised 26 Alport syndrome patients, 15 males and 11 females. Their age ranged from 4 to 16 years (mean ± SD was 8.50 ± 2.877) and 24 were normal controls. Controls criteria are subjects matching age and sex with normal urine analysis, normal kidney function, normal blood pressure, no family history of renal affection, nor any hearing difficulty.

The study protocol has been approved by the Medical Research Ethics Committee of the National Research Center in Cairo, Egypt, following the ethical criteria outlined in the Declaration of Helsinki. Every patient, or their legal guardian, gave their written informed permission. **Inclusion Criteria** of patients were positive family history, presence of persistent hematuria with complement level normal, proteinuria, majority of cases are normotensive, sometimes high blood pressure, hearing loss, eye problems, and renal biopsy revealing picture of Alport syndrome, genetic tests if affordable. Exclusion criteria include other causes of chronic kidney disease, diabetes, and autoimmune diseases.

Patients were recruited from the Clinical Genetics Clinic, Medical Research Center of Excellence, National Research Centre, Egypt. Written informed consent was taken from all the parents. The National Research Centre Ethical Research Committee (Number 013100324 date 26/3/2024) certified that the study’s laboratory procedures and recruitment practices adhered to the Declaration of Helsinki.

All patients undertook regular Alport syndrome workups as a complete medical history, a detailed family history and a three-generation pedigree analysis were done. Clinical examination, urine analysis to search for the presence of hematuria and proteinuria, kidney functions, hearing test by Auditory Brain Response (ABR) looking for sensorineural hearing loss, Ocular examination using Slit Lamp examination, looking at the surface of the cornea and examining the retina searching for perimacular flecks or anterior lenticonus or retinitis pigmentosa. Renal biopsy using Light microscopy and Electron Microscopy. All cases were advised to go for genetic testing. Management of Alport Syndrome patients according to recommendations was Angiotensin converting enzyme inhibition ACEi [12], , Omega-3 Polyunsaturated Fatty Acids [14], , regular audiological and ophthalmological follow-up, screening of at risk relatives [13].

Serum levels of cyclooxygenase-2, Prostaglandin E2, Interleukin 4, and Plasminogen activating inhibitor 1 samples taken early morning and were evaluated in all patients and controls.

### Biochemical analysis

#### Determination of cyclooxygenase-2 (COX-2)

COX-2 was determined by using the ELISA kit (SUNLONG Biotech Co., Ltd), catalog number SL0567 HU) according to the manufacturer’s protocol.

#### Determination of prostaglandin E2 (PGE2)

PGE2 was determined by the ELISA kit (SUNLONG Biotech Co., Ltd), catalog number SL1463 HU) according to the manufacturer’s protocol.

#### Determination of Plasminogen activating inhibitor- 1 (PAI-1)

PAI-1 was determined by the ELISA kit (SUNLONG Biotech Co., Ltd), catalog number SL1407 HU) according to the manufacturer’s protocol.

#### Determination of Interleukin- 4 (IL-4)

IL-4 PAI-1 was determined by ELISA kit (SUNLONG Biotech Co., Ltd), catalog number SL0997 HU) according to the manufacturer’s protocol.

### Statistical analysis

The data were analyzed using the SPSS 20.0 (SPSS, Chicago, IL, USA) software and are expressed as mean ± standard deviation (SD). The results were considered significant at *P* < 0.05. Data analysis was done using the student’s t-test, and Spearman correlation was used to detect the relation between inflammatory markers. A two-sided *p*-value < 0.05 was used to define statistical significance.

## Results

In the studied cohort, there were 15 males and 11 females in a ratio of 1.4:1, with positive consanguinity observed in 14 patients (53.8%), and 9 patients (34.6%) had similarly affected members. The age ranged from 4 to 16 years (mean ± SD was 8.50 ± 2.877). Renal biopsy of all patients revealed thinning of the glomerular basement membrane and focal mesangial glomerulonephritis by Electron Microscopy. Hearing loss was present in 80% of cases, while ocular features were 0% as ocular manifestations present in late stages of renal failure, in our cases only 20% had renal failure in early stage. (Table 1).

Significant increase in serum levels of cyclooxygenase-2, interleukin 4, plasminogen, and prostaglandin E2 compared to the control group. They were 588.68 ± 73.08 in Alport patients versus 369.12 ± 50.28 controls, for interleukin 4 was 42.32 ± 3.49 versus 28.89 ± 3.19 controls, plasminogen activating inhibitor 1 was 846.47 ± 45.433 versus 312.79 ± 40.53 controls, and prostaglandin E2 was 42.57 ± 4.18 versus controls 25.52 ± 4.98 (Table 2). Significant positive correlation between cyclooxygenase-2 and other inflammatory parameters (Table 3).

**Table 1:** Clinical, Laboratory, and other investigations in children with Alport syndrome

Characteristics	Percent (%)
Age (years)	8.50 ± 2.877
Male/Female	15/ 11 (ratio of 1.4:1)
Consanguinity	53.8
Similarly affected members	34.6
Hematuria	100
Proteinuria	40
Hypertension	30
Chronic Renal Failure	25
Hemodialysis	0.00
Renal biopsy Focal mesangial glomerulonephritis	100
Sensorineural hearing loss	77
Ocular manifestations	0.00

**Table 2:** Inflammatory markers in Alport syndrome patients and control

Parameters	Alport syndrome patients	Controls	*P*
Cyclooxygenase-2 (COX-2) (pg/ml)	588.68 ± 73.08	369.12 ± 50.28	0.001
Prostaglandin E2 (pg/ml)	42.57 ± 4.18	25.52 ± 4.98	0.001
Interleukin 4 (pg/ml)	42.32 ± 3.49	28.89 ± 3.19	0.001
Plasminogen activating inhibitor 1 (pg/ml)	846.47 ± 45.433	312.79 ± 40.53	0.001

**Table 3:** Correlation of cyclooxygenase-2 with inflammatory parameters

	*r*	*p*
Prostaglandin E2 (pg/ml)	0.79	0.01
Interleukin 4 (pg/ml)	0.78	0.01
Plasminogen activating inhibitor 1 (pg/ml)	0.86	0.01

## Discussion

The hallmarks of Alport syndrome are hematuria, hypertension, proteinuria, bilateral sensorineural hearing loss, abnormalities of the eyes, and renal failure [15]. The age of our patients ranged from 4 to 16 years (mean ± SD was 8.50 ± 2.877), similarly, Watson et al., 2024 stated that children with Alport syndrome are often diagnosed between the ages of 5 and 10 years [15]. There were 15 males and 11 females in a ratio of 1.4:1, which is comparable to earlier research in which males were more affected than females [15, 16]. This also could be a consequence of the X-linked inheritance of Alport syndrome in 85% of cases. Fourteen patients were the offsprings of consanguineous marriage (53.8%) and 10 patients (34.6) had similarly affected members, this agrees with the findings of Feingold et al., 1985 [17]. All patients presented with hematuria and 23 (88.4%) presented with proteinuria, comparable results were published [18, 19].

One of the most important diagnostic tools for Alport syndrome is a kidney biopsy that was performed for all patients and revealed abnormally Split and laminated glomerular basement membrane (GBM), inflammation all has focal mesangial glomerulonephritis and interstitial fibrosis. This was consistent with the results of Widjaja et al., 2022 [20]. Sensorineural hearing loss for high frequencies has been identified in people with Alport syndrome [3, 21]. In our cohort sensorineural hearing loss was present in 20 patients (77%). Lack of retinopathy or other eye anomalies among our patients may be due to their young age. Previous studies have demonstrated that up to 30% of patients develop retinopathy by the time they reach their fourth decade of life, by the time they have already experienced hearing loss, renal failure, and retinopathy [21].

However, Alport syndrome should still be considered in the differential diagnosis even when retinopathy is absent.

Inflammatory markers were more elevated in patients with low GFR in our study compared to patients with normal GFR, but due to small number of cases this was not significant. This elevation in inflammatory markers with more kidney injury was also clarified in other studies [7].

We surfed the net for research studies revealing role of inflammatory markers on various renal diseases but revealed nothing. A Study on Netrin-1 and Cyclophilin A (CypA) as inflammatory biomarkers for renal injury and Alport syndrome was the first to highlight these crucial biomarkers’ role [3].

Studies have shown that Alport syndrome can be associated with inflammation infiltrating the kidney tissue, potentially contributing to the disease progression [3, 22]. (The expression of COX-2 in the kidney is low in normal physiological conditions, but it becomes significantly higher in response to inflammation and renal fibrosis [23]. While some studies have investigated the role of COX-2 in renal illnesses in general, there hasn’t been much focus on the relationship to Alport syndrome specifically up to this point. In our study, cyclooxygenase-2 levels were higher in Alport patients than in controls; this discrepancy may be attributed to the inflammatory nature of Alport syndrome. Prostaglandins play a key role in the generation of the inflammatory response. Prostaglandin E2 levels were higher in the patients than in the controls; Mutsaers and Nørregaard (2022) found comparable findings [12]. Interleukin 4 is an anti-inflammatory cytokine, that helps regulate the immune system and reduce inflammation. In our study, the patients’ levels of interleukin 4 were predicted to be greater when compared to controls. According to some researchers, IL-4 may provide a therapeutic option for Alport syndrome by reducing inflammation and delaying the course of the illness [24]. Plasminogen-activating inhibitor 1 levels were higher in our report compared to controls. Because plasminogen-activating inhibitor 1 regulates fibrinolysis, elevated levels may exacerbate fibrosis and contribute to the disease progression [11]. Consequently, to successfully stop the progression of renal function loss, more potent therapeutic choices are required. With the enhancement of inflammatory signals to mesangial cells and podocytes, inflammation seems to be recovered [25].

## Conclusion

In this study, we delineated the role of inflammatory markers cyclooxygenase-2, Prostaglandin E2, Interleukin 4, and Plasminogen activating inhibitor 1 in Alport syndrome that are causally connected and has a role in the development and course of Alport disease. This may highlight and speculate an innovative strategy for targeting the creation of safe and efficient anti-inflammatory treatments to inhibit disease progression in Alport syndrome.

### Limitations of study

We were focused by some limitations as small sample size that affected subgrouping and as example clarifying effect of renal affection severity on inflammatory markers.

Time shortage and financial issues limited comparing Alport syndrome cases with other renal diseases presenting with aggressive inflammatory processes that would really enhance the study. Lack of genetic test results is crucially important. The research is a pilot study which adds to the limitations we face.

## Data Availability

Data is provided within the manuscript.
